# Temporal Regulation of Distinct Internal Ribosome Entry Sites of the *Dicistroviridae* Cricket Paralysis Virus

**DOI:** 10.3390/v8010025

**Published:** 2016-01-19

**Authors:** Anthony Khong, Jennifer M. Bonderoff, Ruth V. Spriggs, Erik Tammpere, Craig H. Kerr, Thomas J. Jackson, Anne E. Willis, Eric Jan

**Affiliations:** 1 Department of Biochemistry and Molecular Biology, University of British Columbia, Vancouver, BC V6T 1Z3, Canada; anthony.k.khong@gmail.com (A.K.); jbonderoff@gmail.com (J.M.B.); etammpere@gmail.com (E.T.); kerrc15@gmail.com (C.H.K.); 2 Medical Research Council Toxicology Unit, Leicester LE1 9HN, UK; rvs3@leicester.ac.uk (R.V.S.); tomjacksonhk@gmail.com (T.J.J.); aew5@le.ac.uk (A.E.W.)

**Keywords:** virus, translation, IRES, ribosome, dicistrovirus

## Abstract

Internal ribosome entry is a key mechanism for viral protein synthesis in a subset of RNA viruses. Cricket paralysis virus (CrPV), a member of *Dicistroviridae*, has a positive-sense single strand RNA genome that contains two internal ribosome entry sites (IRES), a 5′untranslated region (5′UTR) and intergenic region (IGR) IRES, that direct translation of open reading frames (ORF) encoding the viral non-structural and structural proteins, respectively. The regulation of and the significance of the CrPV IRESs during infection are not fully understood. In this study, using a series of biochemical assays including radioactive-pulse labelling, reporter RNA assays and ribosome profiling, we demonstrate that while 5′UTR IRES translational activity is constant throughout infection, IGR IRES translation is delayed and then stimulated two to three hours post infection. The delay in IGR IRES translation is not affected by inhibiting global translation prematurely via treatment with Pateamine A. Using a CrPV replicon that uncouples viral translation and replication, we show that the increase in IGR IRES translation is dependent on expression of non-structural proteins and is greatly stimulated when replication is active. Temporal regulation by distinct IRESs within the CrPV genome is an effective viral strategy to ensure optimal timing and expression of viral proteins to facilitate infection.

## 1. Introduction

Viral protein synthesis is an essential process in all viral life cycles. As such, viruses have adapted diverse strategies to recruit the host ribosome and the translational machinery. Numerous positive strand RNA viruses use a strategy whereby infection leads to an inhibition of host translation concomitant with an increase in viral protein synthesis [1]. One of the best-studied examples of this switch from host to viral protein synthesis is during poliovirus infection: the translation factors eIF4G and poly A binding protein are cleaved by virally-encoded proteases thereby shutting off cap-dependent translation [2,3,4]. By contrast, the poliovirus internal ribosome entry site, IRES, recruits the ribosome using a subset of translation factors and IRES trans-acting factors (ITAFs) to direct cap-independent viral protein synthesis [5]. Therefore, the strategies that underlie the switch from host to viral protein synthesis such as the signalling pathways that target translation factors and the mechanisms that direct viral protein expression are fundamental to virus infection.

**Figure 1:**
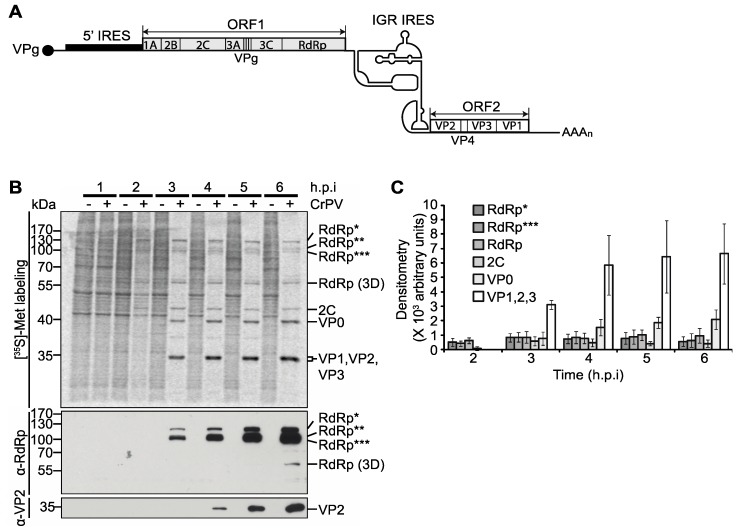
Viral non-structural and structural protein expression in CrPV-infected S2 cells. (**A**) Schematic of the genome arrangement of CrPV; (**B**) Autoradiography of pulse-labelled protein lysates from S2 cells either mock- or CrPV-infected (MOI 10) resolved on a 12% SDS-PAGE gel. The protein lysates were collected from S2 cells at the indicated times hours post-infection (h.p.i.) and metabolically labelled with [^35^S]-Met/Cys for twenty minutes at the end of each time point (top). The identities of specific CrPV viral proteins, as shown on the right of the gel, were previously described [7,8] and determined by immunoblotting with α-RdRp and α-VP2 peptide antibodies or predicted by molecular weight (below); (**C**) Raw densitometric quantitation of [^35^S]-Met/Cys pulse-labelled RdRp*, RdRp**, RdRp***, 2C, VP0, and VP1, 2, and 3 during CrPV infection from three independent experiments (±s.d.). RdRp*, RdRp**, and RdRp*** denote polyproteins containing RdRp at the approximate sizes of 120 kDa, 105 kDa, and 100 kDa respectively.

The *Dicistroviridae* family uses an interesting strategy for viral protein synthesis: the monopartite plus strand ~9 kb RNA genome contains two IRESs, the 5′untranslated region (5′UTR) and the intergenic (IGR) IRES that direct translation of two open reading frames (ORFs) encoding the non-structural and structural proteins, respectively (Figure 1A) [6]. Infection of Drosophila S2 cells by the dicistrovirus *Cricket paralysis virus* (CrPV) results in the rapid shut off of host protein synthesis concomitant with preferential viral protein synthesis involving supramolar expression of the structural proteins compared to the non-structural proteins [7,8,9]. The significance and the regulation of the CrPV IRES translation during infection have not been examined in detail.

The 5′UTR- and IGR IRES can support translation in a number of translation systems both *in vitro* and *in vivo*, including in yeast, insect and mammalian extracts and cells [10,11,12,13]. The IGR IRES has been studied extensively [14,15,16,17,18,19]; the IGR IRES directly recruits the ribosome without the aid of translation initiation factors and initiates translation from a non-AUG codon [14]. Biochemical and structural studies have revealed that the IGR IRES adopts distinct structural domains that enable the IRES to occupy the intersubunit core of the ribosome in order to gain access to the ribosomal A and P sites [15,16,20,21,22,23]. Indeed, the CrPV pseudoknot I domain of the IGR IRES structurally mimics the anticodon of a tRNA [15,22,23]. Recent cryo-EM studies have demonstrated that the PKI domain first occupies the A site followed by translocation to the P site and thereby leaving the A site unoccupied for delivery of the initial aminoacyl-tRNA [22,23]. By contrast, studies on the mechanism of the 5′UTR IRES have been limited [13,24]; using a reconstitution approach, Terenin *et al.* (2005) showed that eIF1, eIF2, and eIF3 are required for 5′UTR IRES dependent translation of a related dicistrovirus *Rhapdopsilum padi virus* (RhPV) [24]. Differences in the mechanism of the CrPV 5′UTR and IGR IRESs likely contribute to distinct expression levels of non-structural and structural proteins. Supporting this, 5′UTR IRES translation is weaker than IGR IRES translation [9] and that IGR IRES translation is stimulated during CrPV infection using transfection approaches of reporter RNAs [25]. However, the significance of this strategy by dicistroviruses is not well understood. Many RNA viruses control the timing of viral protein expression in order to facilitate viral replication early in infection and viral packaging later in infection [26]. For example, in *Alphavirus* infections, non-structural proteins are expressed prior to structural protein synthesis in order to coordinate replication and viral assembly temporally [26].

In this study, using a series of approaches including radioactive pulse-labelling, reporter construct assays and ribosome profiling, we demonstrate that the 5′UTR and IGR IRES activities are temporally regulated during CrPV infection. Specifically, IGR IRES translation is delayed until later times in infection when compared to 5′UTR IRES translation. The delay is not due to a general decrease in host translation, an increase in eIF2α phosphorylation during infection or a decrease in host transcription. Using a novel CrPV replicon that uncouples the effects of replication and translation, our data suggest that non-structural viral proteins may be partly responsible for the increase in IGR IRES dependent translation during infection. In summary, CrPV uses an IRES-dependent temporal regulation strategy in order to ensure optimal expression of structural and non-structural proteins and to facilitate the timing of events during the viral life cycle.

## 2. Materials and Methods

### 2.1. Cell Culture, Virus Infection, and Viral Titers

Drosophila S2 cells were maintained in Shields and Sang M3 insect media (Sigma-Aldrich, Oakville, ON, Canada) supplemented with 10% fetal bovine serum (SSM3) at 25 °C as described previously [7]. For virus infections, S2 cells were washed with PBS, then incubated with CrPV for the indicated MOI for 30 min and subsequently, washed with PBS and resuspended with SSM3. Viral titers were determined by a fluorescence-focusing assay as described [7].

### 2.2. Reporter Construct and CrPV2 Replicon

The construction of the CrPV minigenome reporter was described in Kerr *et al.* (2015) [27]. For the CrPV2(Fluc), CrPV2(Fluc)-ORF1^stop^, and CrPV2(Fluc)-mutRdRp replicons, standard recombinant DNA technology was used to construct these plasmids. Unique restriction sites that facilitated insertion of the firefly luciferase gene into the CrPV-2 clone [27] were introduced by site-directed mutagenesis. Specifically, an XhoI site was introduced by substituting two nucleotides within ORF2 that encodes VP2 and a SacII site was introduced by adding the restriction sequence, 5′-CCGCGG-3′, after ORF2 in CrPV2. The primer pairs used for introducing XhoI are 5′-ATGAAGATAAAAGACTCGAGTCAGAACAGAAAGAAATTGTACATT-3′ and 5′-CTCGAGTCTTTTATCTTCATAAACAAGAAAATTCACACATTGAAA-3′ and the primer pairs used for introducing SacI are 5′-TAGCACGCGCCTAACCGCGGCTAACTATTTGCTTTGTATTTTAAG-3′ and 5′-CCGCGGTTAGGCGCGTGCTACATTTACTAAAGGTGGAACTCCAAG-3′. Mutations were sequenced to verify. To generate CrPV2(Fluc), firefly luciferase gene was amplified from the CrPV minigenome reporter with primers containing XhoI and SacII flanking the luciferase gene (5′-AAACTCGAGATGGAAGACGCCAAAAACATA-3′ and 5′-AAACCGCGGTTACACGGCGATCTTTCCGCC-3′). The CrPV-2 plasmid containing XhoI and SacII and the amplified firefly luciferase gene were digested with XhoI and SacII restriction enzymes. The backbone of the CrPV-2 plasmid was then gel purified (Qiagen, Hilden, Germany). Subsequently, the PCR-purified (Qiagen) digested firefly luciferase gene was combined in a ligation reaction with the CrPV-2 backbone template as described in the manufacturer’s protocol (Thermo Fisher Scientific, Waltham, MA, USA). After transforming the ligation reaction, DH5α bacteria colonies were picked and insertion of the firefly luciferase gene into the CrPV-2 clone was sequenced verified. To generate CrPV2(Fluc)-ORF1^stop^ and CrPV2(Fluc)-mutRdRp replicons, site directed mutagenesis was performed. For CrPV2(Fluc)-ORF1^stop^, the primers used for site-directed mutagenesis were 5′-ATTCTTACAATGTGATCATGTCTTTTTAATAAACAAACAACAACGCAACCAACAAC-3′ and 5′-GTTGTTGGTTGCGTTGTTGTTTGTTTATTAAAAAGACATGATCACATTGTAAGAAT-3′. For CrPV2(Fluc)-mutRdRp, the primers used for site directed mutagenesis were 5′-GATGATAAGCTACGGAGCTGCCAATTGCCTAAATATTT-3′ and 5′-AAATATTTAGGCAATTGGCAGCTCCGTAGCTTATCATC-3′. Clones were fully sequenced for verification.

### 2.3. RNA Transfection and Luciferase Assay

*In vitro* transcription reactions using T7 RNA polymerase was performed as described previously [25]. RNA was polyadenylated (CellScript, Madison, WI, USA), purified (RNeasy kit, Qiagen) and the integrity of the RNA was determined by visualizing it on an agarose gel.

Transfection of 1 µg RNA (lipofectamine 2000, Invitrogen) into S2 cells (1.5 × 10^6^ cells per mL; 12-well plates) was performed as described by the manufacturer (Invitrogen). Cells were harvested with passive lysis buffer (Promega, Madison, WI, USA) and assayed for luciferase activity (Promega) using a microplate luminometer (Berthold Technologies, Centro LB 960, Bad Wildbad, Germany).

### 2.4. Western Blotting

As described previously [7], cells were washed once with 1× PBS and harvested in lysis buffer (20 mM HEPES, 150 mM NaCl, 1% Triton X-100, 10% glycerol, 1 mM EDTA, 10 mM tetrapyrophosphate, 100 mM NaF, 17.5 mM β-glycerophosphate, and protease inhibitors (Roche, Basel, Switzerland)). Equal amounts of lysates, as determined by Bradford assay, were resolved on a 10%–15% acrylamide SDS-PAGE gel. For western blotting, the following antibodies were used: 1:10,000 CrPV VP2 antibody (PL Laboratories, Port Moody, BC, Canada), 1:10,000 CrPV RdRP antibody (PL Laboratories), or 1:500 phospho-eIF2α antibody (Cell Signaling, Whitby, ON, Canada).

### 2.5. [^35^S]-Met/Cys Pulse-Labelling

As described previously [7], 5 × 10^6^ S2 cells were pulse labelled with 250 µCi [^35^S]-Met/Cys (Perkin-Elmer, Waltham, MA, USA) for twenty minutes, washed with 0.5 mL cold PBS twice and harvested with 50 µL lysis buffer. Equal amounts of lysates were resolved with SDS-PAGE. The gel was dried and radioactive bands were analyzed by a phosphorimager (Amersham Pharmacia Biotech, Amersham, UK). Densitometric analysis on the pulse-labelled bands was performed using ImageJ software [28].

### 2.6. Immunoprecipitation

S2 lysates were incubated with a fixed amount of CrPV RdRp or VP2 rabbit polyclonal primary antibodies respectively in immunoprecipitation lysis buffer (50 mM Tris-HCL, pH 7.5, 150 mM NaCl, 1% Nonidet P40, 0.5% sodium deoxycholate, and protease inhibitors (Roche)) for one hour at 4 °C with gentle agitation. Fifty microliters of protein G-agarose (Roche) were incubated with the reaction overnight at 4 °C. Mixtures were centrifuged at 2000 rpm for one minute and the precipitate was washed twice with the immunoprecipitation lysis buffer, twice with wash buffer 1 (50 mM Tris-HCL, pH 7.5, 500 mM NaCl, 0.1% Nonidet P40, and 0.05% sodium deoxycholate), and once with wash buffer 2 (50 mM Tris-HCL, 0.1% Nonidet P40, and 0.05% sodium deoxycholate). The precipitate was resuspended in 50 µL SDS loading dye and removed from the beads by heating at 95 °C for ten minutes and centrifugation at 13,200 rpm. The samples were resolved by SDS-PAGE. The gel was dried and imaged with a phosphoimager (Amersham Pharmacia Biotech). Densitometric analysis on the pulse-labelled bands was performed using ImageJ software [28].

### 2.7. dsRNA-Mediated Knockdown

The protocol for dsRNA-mediated knockdown in Drosophila S2 cells was adapted from Cherry *et al.* (2005) [29]. Snapdragon [30], a web-based tool, was used to identify appropriate primers for amplifying fragments of genes that are intended for knockdown while minimizing off target effects. Specifically, the PCR products of dPERK (437–853 nucleotides, NM_141281) and dGCN2 (492–1034 nucleotides, NM_057882) was RT-PCR amplified from total RNA of Drosophila S2 cells using primers that contain the T7 promoter at both ends. dPERK was PCR amplified using: 5′-ACTGACTAATACGACTCACTATAGGGCTGGGCACCCAACTACTGAT-3′ and 5′-ACTGACTAATACGACTCACTATAGGGAACGGAAACACCGTATGAGC-3′. dGCN2 was PCR amplified using: 5′-ACTGACTAATACGACTCACTATAGGGGAGACTCCTCGAACGGACTG-3′ and 5′-ACTGACTAATACGACTCACTATAGGGGGAAGTAGAGCGTCTCCGTG-3′. The PCR products were used as templates in *in vitro* transcription reactions using T7 RNA polymerase. dsRNA was purified and the integrity was verified by gel analysis. dsRNA (13 µg/million cells) was incubated with S2 cells in serum-free SSM3 media for one hour, followed by the addition of complete SSM3 media.

### 2.8. S2 Translation Extracts and *in Vitro* Translation Assay

The protocol for S2 translation extracts was adapted from Brasey *et al.* (2003) and Roy *et al.* (2004) [31,32]. Specifically, 2.0 × 10^9^ mock-infected or 6 h.p.i. CrPV-infected (MOI 10) S2 cells were pelleted at 1000 g for eight minutes, washed once with PBS and resuspended in 2 mL hypotonic buffer (10 mM HEPES-KOH (pH 7.4), 10 mM KOAc, 0.5 mM MgOAc, 1 mM DTT). After incubation in hypotonic buffer on ice for five minutes, the cells were lysed by extrusion 25 times through a 23-gauge needle. The extracts were then adjusted to 50 mM KOAc. The supernatant (S2 translation extract) was collected upon clearing all cell debris by centrifugation at 16,000 g for five minutes at 4 °C, aliquoted, and stored at −80 °C.

Prior to use, the S2 translation extract was supplemented with 0.2 U creatine phosphokinase/µL. Each translation assay was carried out in a final volume of 10 µL containing 6.5 µL S2 translation extract, 2 µL 5× master mix (10 µM complete amino acids, 40 mM creatine phosphate, 100 mM HEPES-KOH (pH 7.6), 5 mM ATP, 1 mM GTP, 2.5 mM spermidine, 500 mM KOAc, and 5 mM MgOAc), 0.5 µL Ribolock RNase Inhibitor (40 U/µL, Thermo Fisher Scientific), and 9 nM template RNA for thirty minutes at 30 °C. After incubation, half of the reaction was assayed for luciferase activity (Promega).

### 2.9. Ribosome Profiling

The ribosome profiling experiment was adapted from Ingolia *et al.* (2009) [33]. Specifically, Drosophila S2 cells were infected with CrPV (MOI 10) or mock-infected. At the indicated time points, samples were subject to total RNA extraction using Trizol (Thermo Fisher Scientific) for RNAseq analysis or were subject to RNA isolation from ribosomes. For the latter, cells were first treated with cycloheximide (100 μg/mL), pelleted, washed with PBS containing cycloheximide (100 μg/mL), pelleted again, and lysed with 1× RPF buffer (300 mM NaCl, 15 mM MgCl2, 15 mM Tris-HCl pH 7.5, 2 mM DTT, and 100 μg/mL cycloheximide) containing 1% Triton ×100 and 500 U/mL RNaseIn on ice for ten minutes. Afterwards, the lysates were spun at 10,000 rpm for ten minutes at 4 °C. The supernatant was collected and is now referred to as S10 lysates. S10 lysates were treated with 2 U/µL RNase I (Thermo Fisher Scientific) for thirty minutes at room temperature. The S10 lysate was loaded onto a 10%–50% sucrose gradient containing 15 mM Tris-HCl pH 7.5, 300 mM NaCl, 15 mM MgCl2, 2 mM dithiothreitol, 100 µg/mL cycloheximide, and 0.1 U/µL SUPERase*in (Ambion) and ultracentrifuged for three hours at 35,000 rpm. Gradients were fractionated using a Brandel syringe pump and ISCO UV detector; the top two monosome peak fractions were extracted twice with acid phenol and ethanol precipitated. The RNA collected from these monosome peak fractions are now referred to as ribosome protected fragments (RPFs). The RPFs were depleted of 2S rRNA by subtractive hybridization [34]. Specifically, RPFs were incubated with 100 pmol of biotinylated DNA oligo (TACAACCCTCAACCATATGTAGTCCAAGCA, Integrated DNA Technologies, Coralville, IA, USA) at 65 °C for two minutes and then at 37 °C for fifteen minutes in 2× SSC (300 mM NaCl, 30 mM sodium citrate). An excess of streptavidin-coated beads was added and incubation continued on a rotator for fifteen minutes at 25 °C. The supernatant was recovered and precipitated by ethanol. Finally, the precipitated RPF RNA was gel-excised using 27 and 33 nucleotides RNA oligonucleotide markers.

Total RNA was prepared for RNAseq using the SOLiD Total RNAseq kit (Thermo Fisher Scientific). The gel-excised RPF RNA was prepared for RNAseq using the Small RNA Library Procotol from the SOLiD Total RNAseq Kit (Applied Biosystems). Libraries were sequenced on the SOLiD 4 platform.

### 2.10. Calculations of Translational Efficiency

The raw csfasta and qual files produced by each run of SOLiD 4 were converted to xsq files using the XSQ Tools package from life technologies™ (Thermo Fisher Scientific). Reads within these xsq files were mapped to the CrPV genome using “genomic resequencing” mapping within LifeScope™ from life technologies™. Reference files for mapping were created using the sequence and annotations provided in NC_003924.1 (Wilson *et al.* (2009)) [35]. A mapping quality threshold of 8 was used. Each mapped read was labelled depending on its position with relation to the two ORFs of the CrPV genome. The raw numbers of reads were converted to rpM (reads per million) to normalize for the different library sizes between SOLiD runs. Translational efficiency was calculated using rpM values for the ribosome protected fragments (RPF) and for the Whole Transcriptome set (WT), and is RPF divided by WT. To find any translational efficiency changes across the two ORFs, the CrPV genome was split into five sections, namely 5′UTR, ORF1, IGR, ORF2, and 3′UTR. A *t*-test statistical analysis was performed to determine if there were significant changes in translational efficiency values.

### 2.11. Uridine Labelling of RNA

S2 cells (5 × 10^6^) were pretreated with actinomycin D for fifteen minutes, followed by the addition of 5 µCi of [^3^H]-uridine (Perkin Elmer) for fifteen minutes. Total RNA was extracted (Trizol, Thermo Fisher Scientific). Equal amounts of RNA were separated in a denaturing agarose gel and transferred to nylon membrane. Levels of radioactivity were detected by phosphorimaging and quantified using ImageQuant software.

## 3. Results

### 3.1. Translation of CrPV ORF1 and ORF2 during CrPV Infection

Although it is well established that viral structural proteins are expressed in molar excess over the viral non-structural proteins during CrPV infection [7,8,9], we carefully re-examined viral protein synthesis in CrPV-infected S2 cells by metabolic [^35^S]-Met/Cys pulse-labelling at different times after infection. As observed previously [7,8,9], host translation decreases dramatically two to three hours post infection (h.p.i) concomitant with the synthesis of CrPV proteins (Figure 1B). The processed and unprocessed viral structural and non-structural proteins were annotated based on previous studies [7,8,9] and from immunoblots using antibodies that recognize RNA-dependent RNA polymerase (RdRp) and VP2 structural protein (Figure 1B) [7]. Using an RdRp antibody for Western blot analysis, we detected unprocessed RdRp non-structural proteins at 120 (RdRp*), 105 (RdRp**), and 100 kDA (RdRp***) and the mature 3D at 60 kDa. A close inspection of the pulse-labelled proteins showed that the non-structural proteins such as the unprocessed 120 kDa RdRp and 2C are detected first at two h.p.i. whereas the structural proteins are not detected until after three h.p.i. (Figure 1B), which is similar to that observed previously [7,9]. Quantitation of the viral proteins confirmed that the non-structural proteins are first expressed by two h.p.i. and increase steadily at later times of infection and that the viral structural proteins are not detected until three to four h.p.i. (Figure 1C). We note that quantitation of the pulse-labelled non-structural proteins at two h.p.i. may be masked due to ongoing host protein synthesis at this time of infection (Figure 1B). To detect the non-structural proteins more accurately, we monitored synthesis by immunoprecipitation of pulse-labelled structural and non-structural proteins from CrPV-infected cell lysates (Figure S1). Specifically, we pulse-labelled cells for one hour followed by lysis and immunoprecipitation using α-RdRP and α-VP2 antibodies. We initially optimized the immunoprecipitation conditions to ensure that the antibody is saturating by incubating increasing amounts of [^35^S]-Met/Cys labelled lysates with a constant amount of antibody (Figure S1A,B). Immunoprecipitation with the α-RdRp antibody pulled down unprocessed and mature RdRp from infected lysates whereas the α-VP2 immunoprecipitated all four structural proteins, likely as a packaged virion (Figure S1A,B). At two h.p.i., non-structural proteins were readily detected by α-RdRp pulldowns. In contrast, the structural proteins were not detected until three h.p.i. (Figure S1C,D). Furthermore, whereas the pulse-labelled RdRp is relatively constant throughout infection, the structural protein immunoprecipates increase significantly at later times of infection, supporting the notion that translation of the two ORFs are distinctly and temporally regulated during infection (Figure S1C,D). In summary, the pulse-labeling experiments suggest that translation of the downstream ORF driven by the IGR IRES is delayed until three to four h.p.i.

### 3.2. Ribosome Profiling of Viral RNA in CrPV-Infected S2 Cells

**Figure 2:**
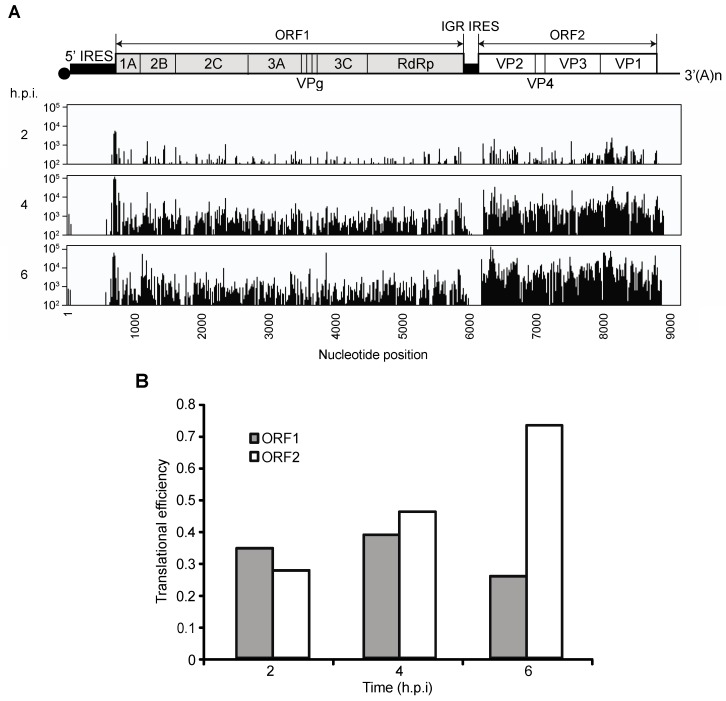
Ribosome profiling of the CrPV RNA in infected S2 cells. (**A**) CrPV RNA sequence coverage (*x*-axis) in rpM from the ribosome-associated fractions (*y*-axis) at two, four and six h.p.i (MOI 10). The coverage, as indicated, is aligned to the viral genome shown above; (**B**) Translational efficiency was calculated by dividing reads per million values for the ribosome protected fragments (RPF) by the Whole Transcriptome set (WT). The TE was determined from the average of two independent experiments.

The distinct temporal expression of non-structural and structural proteins may be explained by one of two broad hypotheses. (1) The increase in structural protein synthesis can be described by some form of passive regulation during infection—a combination of ribosome availability upon host translational repression, different intrinsic affinities for ribosomes and requirements for translation initiation factors between the 5′IRES and the IGR IRES and/or an increase in viral RNAs; (2) alternatively, the increase in viral structural protein synthesis may be driven by some active form of regulation during infection; in other words, IGR IRES translation may be activated or inhibited, possibly by protein factors binding to the IGR IRES and/or by changes in the structure of IGR IRES during infection. To address these two broad hypotheses, we examined IRES activities indirectly by ribosome profiling. Ribosome profiling is a next-generation sequencing-based approach that identifies ribosome occupancy across mRNAs at high nucleotide resolution [33]. RNAseq analysis, which is performed in parallel, takes into account for mRNA abundance, thus the translational efficiency of each mRNA can be calculated. We measured ribosome occupancy on the CrPV RNA at 2, 4 and 6 h.p.i (Figure 2A). Ribosome protected fragments (RPF) were found predominantly within the two main ORFs, and as expected very little RPFs were found within the IGR and 3′UTR. To determine the TE of each ORF, we divided the number of ribosome-protected footprints (RPFs) for each ORF to the average read frequency across the viral RNA genome. At two h.p.i, the translational efficiencies of ORF1 and ORF2 are approximately equal (Figure 2B), which suggests that both ORFs are translated similarly. While the translational efficiency of ORF1 remains relatively constant as infection progresses, the translational efficiency of ORF2 increases by approximately 2.5 fold over the course of infection (Figure 2B). These results suggest that the increased ribosome occupancy on ORF2 is a reflection of increased translation by the IGR IRES at later times of infection.

### 3.3. IRES Translation Using CrPV Minigenome Reporter Analysis

**Figure 3:**
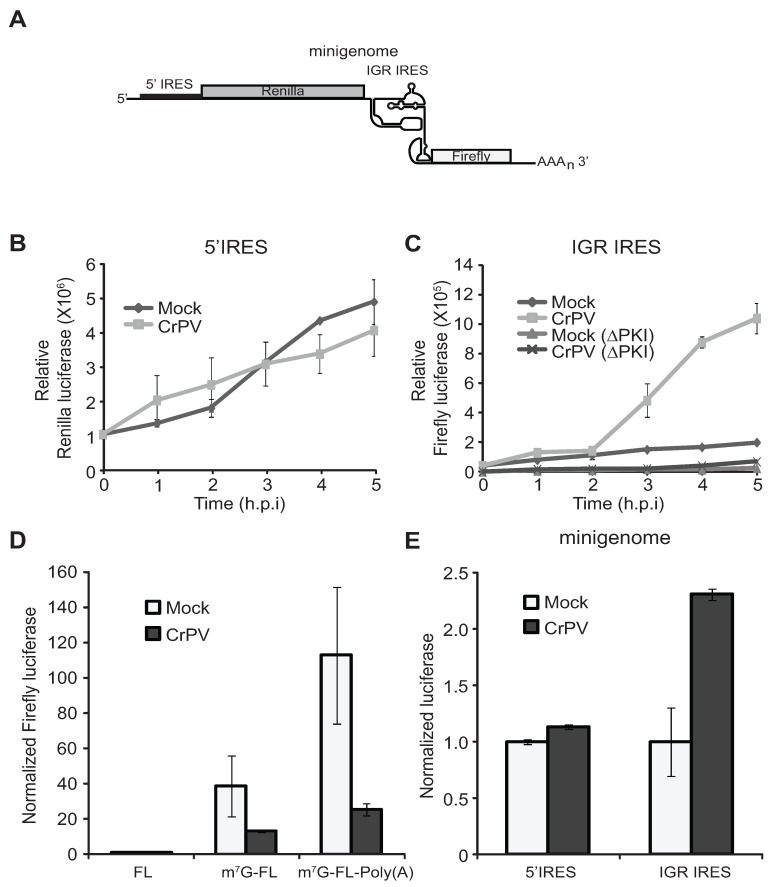
CrPV 5′UTR and IGR IRES translation. (**A**) Schematic of the CrPV minigenome; (**B**,**C**) S2 cells were transfected with the CrPV minigenome RNA one hour prior to mock or CrPV infection. Cells were harvested at the indicated time points and assayed for (**B**) Renilla or (**C**) firefly luciferase activity. A mutation within the IGR IRES (ΔPKI), which disrupts pseudoknot I of the IRES, was used to validate that IGR IRES activity was monitored; (**D**) Monocistronic firefly RNA (FL), 5′-capped monocistronic firefly RNA (m^7^G-FL), and 5′-capped and polyadenylated monocistronic firefly RNA were incubated in S2 translation extracts for thirty minutes and assayed for firefly luciferase activity. The results are normalized to FL incubated in mock extracts; (**E**) Minigenome RNA was incubated in S2 translation extracts from mock or infected cells for thirty minutes and assayed for Renilla and firefly luciferase activities. The results are normalized to mock extracts. (**D**,**E**) Averages are shown from at least three independent experiments (±s.d.).

The ribosome profiling and pulse-labelling experimental results suggest that translation of the CrPV ORFs is regulated. However, it is possible that the increase in ORF2 expression at 3–4 h.p.i. may be reflected in the rapid increase in viral RNA levels due to replication [7]. To uncouple the effects of viral translation from replication, we directly examined 5′UTR and IGR IRES translation using a CrPV reporter minigenome construct whereby the viral ORF1 and ORF2 are replaced with Renilla and firefly luciferase gene, respectively (Figure 3A) [27]. Therefore, Renilla and firefly luciferase activities are used to monitor the 5′UTR and IGR IRES-mediated translation, respectively.

*In vitro* transcribed minigenome RNA was transfected into S2 cells, which were then infected with CrPV an hour later. Previously, we showed that this approach resulted in luciferase activity that increases linearly in the first six hours, indicating that the reporter RNA is engaged in translation during this time [25]. Cells were harvested at 1–5 h after infection and assayed for luciferase activity (Figure 3B,C). As expected, in mock-infected cells transfected with the minigenome RNA, Renilla luciferase activity increased over time in the first five hours after transfection. In CrPV-infected cells, Renilla luciferase activity increased over time at a similar rate as in mock-infected cells, suggesting that 5′UTR IRES translation is relatively constant during infection (Figure 3B). In contrast, firefly luciferase activity accumulated gradually early in infection and sharply increased after two h.p.i., suggesting that IGR IRES translation is delayed at early times of infection and is stimulated at later time points (Figure 3C). To confirm that IGR IRES translation is being monitored, we used a mutant minigenome reporter (ΔPK1) [19] that contains mutations in pseudoknot I that are known to ablate IGR IRES activity. Firefly luciferase activity was severely inhibited in cells transfected with the ΔPK1 minigenome reporter (Figure 3C).

To further confirm that these effects are at the translational level, we compared IRES activity in S2 translation extracts from cells that were mock infected or CrPV-infected at 6 h.p.i. Incubation of monocistronic firefly luciferase reporter RNA that is 5′-capped (m^7^G-FLuc) or 5′-capped and polyadenylated (m^7^G-FLuc-Poly(A)) in S2 extracts resulted in a 38 fold and a 133 fold increase in translation, respectively, as compared to an uncapped, nonpolyadenylated reporter RNA (FL) (Figure 3D), thus showing that the S2 extract supports cap- and poly (A)-dependent translation. In CrPV-infected extracts, a relatively lower levels in luciferase activities were observed with m^7^G-FL and m^7^G-FL-poly(A) RNA, respectively (Figure 3D), consistent with CrPV infection leading to inhibition of host translation [7,8,9]. To address if IGR IRES activity differs in extracts from mock infected or infected cells, we incubated the minigenome RNA in these extracts (Figure 3A) and assayed for luciferase activities. Similar to the *in vivo* results, the CrPV-infected extracts displayed higher IGR IRES activity (Figure 3E). On the other hand, the 5′-UTR IRES activity is similar in mock and infected extracts (Figure 3E). In summary, these results demonstrate that the CrPV 5′UTR and IGR IRESs are differentially and temporally regulated during CrPV infection.

### 3.4. Role of Transcriptional Shutoff in Viral Protein Synthesis under CrPV Infection

**Figure 4:**
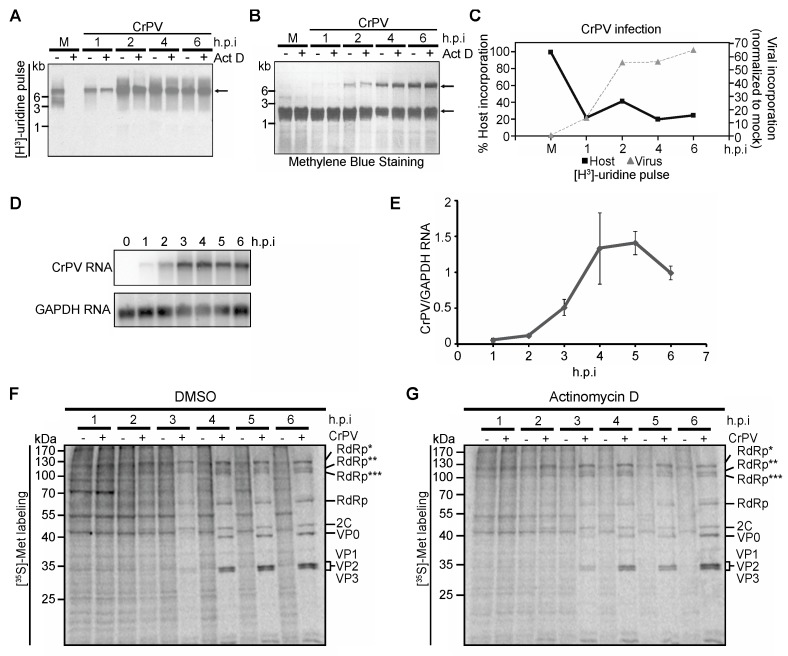
(**A**) Mock- and CrPV-infected S2 cells were pulse-labelled with [^3^H]-uridine for ten minutes at the indicated hours post infection (h.p.i.) before harvesting. Where indicated, the cells were left untreated or treated with actinomycin D (Act D, 5 µg/mL) for fifteen minutes prior to pulse-labeling. RNA was extracted, loaded on a denaturing agarose gel, transferred to a nylon membrane, and analyzed by phosphorimager analysis. Representative autoradiogram and a corresponding methylene blue stain are shown in (**A**,**B**), respectively; (**C**) Quantitation of host and viral transcription rates in CrPV-infected cells. The rate of host transcription at each time point was calculated using the formula [(Total radioactivity in lane − Act D) − (Total radioactivity in lane + Act D)] / (Total radioactivity in lane − Act D). The rate of host transcription was normalized to that in mock-infected cells given as 100%. The rate of viral transcription was calculated at each h.p.i. by using the formula [(Total radioactivity in lane + Act D) / (Total radioactivity in lane – Act D)]. The rate of viral transcription was normalized to that in mock-infected cells given as 0%. Shown are representative gels and averages from at least 2 independent experiments; (**D**) Viral RNA levels were detected by Northern blot analysis; (**E**) Viral RNA levels were quantified using ImageQuant and plotted to show levels of viral RNA/GAPDH RNA at different time points post infection; (**F**,**G**) Autoradiography of pulse-labelled protein lysates resolved on a 12% SDS-PAGE gel from S2 cells mock- or CrPV-infected (MOI 10) and treated with DMSO (**F**) or 50 nM Act D (**G**) three hours prior to infection. RdRp*, RdRp**, and RdRp*** denote polyproteins containing RdRp at the approximate sizes of 120 kDa, 105 kDa, and 100 kDa, respectively. Averages are shown from at least three independent experiments (±s.d.).

A number of viruses inhibit transcription and/or translation during infection. We hypothesize that these widespread disruptions in cellular homeostasis may have an effect on IGR IRES translation. To examine this, we first tested if cellular transcription is affected during CrPV infection, which may be responsible for changes in IGR IRES-dependent translation. Several studies have examined the changes in transcriptomes during dicistrovirus infection in Drosophila S2 cells and in *Drosophila melanogaster* [36,37,38,39]. In general, dicistroviruses repress the transcription of host genes [39]. We verified that CrPV infection repressed cellular transcription by pulse labelling mock and CrPV-infected S2 cells with [^3^H]-uridine for 15 min at specific time points after infection (Figure 4A). RNA was extracted, resolved on an agarose gel, and transferred to a nylon membrane. Methylene blue staining readily detected the 18S/28S rRNA, which migrated at a size of ~2 kb, confirming that the RNA is intact (Figure 4B). In Drosophila, the mature 28S rRNA undergoes cleavage producing two products of approximate size to each other that coincides with the size of the 18S rRNA [40]. Labeling with [^3^H]-uridine in mock-infected cells produced two predominant radiolabelled bands that migrated at ~8 kb and ~5 kb (Figure 4A). Because we only labelled with [^3^H]-uridine for a short period, these labelled RNAs likely represent immature, unprocessed rRNA precursors. As expected, treatment of mock-infected cells with actinomycin D completely inhibited host transcription (Figure 4A). CrPV infection at an MOI of 10 resulted in labeling of a ~9 kb RNA, which increased during the course of infection demonstrating viral replication (Figure 4A). To distinguish between host and viral transcription, host transcription was inhibited by treating cells with actinomycin D (5 µg/mL) prior to [^3^H]-uridine-labeling. As predicted, radiolabelled viral RNA was only detected in actinomycin D-treated infected cells (Figure 4A,C). Viral RNA synthesis increased during the first 4 h of infection before levelling off and correlated with the increase in viral RNA as measured by Northern blot analysis (Figure 4D). In contrast, host transcription was rapidly inhibited within one hour of infection (~80% inhibition) and remained shut off during infection (Figure 4A,C). Host transcription was calculated by subtracting the amount of [^3^H]-uridine labelled RNA in cells treated with actinomycin D from that in cells without actinomycin D. The quantitations of both viral and host transcription is shown in Figure 4C. Since CrPV infection inhibits host transcription, we tested if this effect impacts the temporal regulation of CrPV protein synthesis. We pretreated cells in the absence or presence of actinomycin D for three hours prior to infection and compared the rate of viral protein synthesis in S2 cells by [^35^S]-Met/Cys pulse labelling. If shutoff of transcription is necessary for the enhanced structural protein synthesis at three h.p.i, IGR IRES translation may be prematurely stimulated. No significant changes in viral non-structural and structural protein synthesis were observed between cells pretreated with actinomycin D, suggesting that host transcriptional shutoff does not contribute significantly to the temporal regulation of viral ORF protein synthesis (Figure 4F,G).

### 3.5. Role of eIF2α Phosphorylation in Viral Protein Synthesis under CrPV Infection

**Figure 5:**
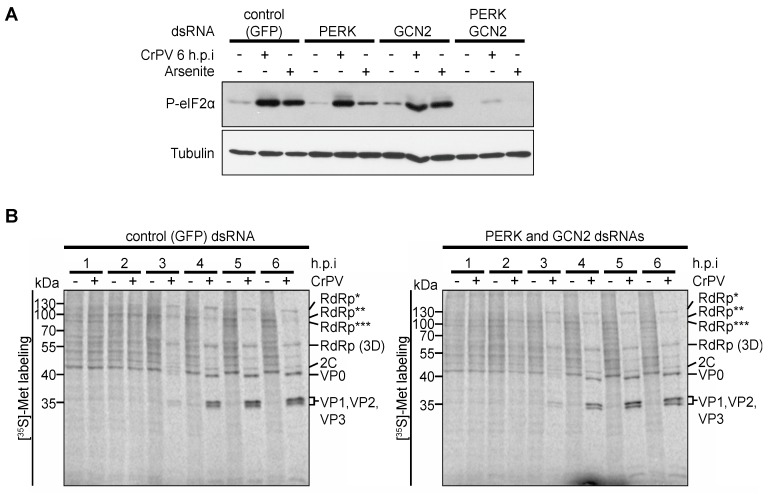
Role of dPERK and dGCN2 eIF2α kinases in CrPV-infected S2 cells. (**A**) Immunoblots (anti-phospho-eIF2α (P-eIF2α) and anti-Tubulin antibodies) of lysates from cells treated with control (GFP) dsRNAs or singly dPERK or dGCN2 dsRNAs or both dPERK/dGCN2 dsRNAs for 48 h followed by arsenite (500 μM) treatment or infection with CrPV (MOI 10); (**B**) [^35^S]-Met/Cys pulse-labelled protein lysates from control or dPERK and dGCN2 dsRNA-treated S2 cells that were either mock or CrPV-infected (MOI 10) for the indicted times (h.p.i.) were resolved on a 12% SDS-PAGE gel. Shown are representative autoradiographs from at least three independent experiments. RdRp*, RdRp**, and RdRp*** denote polyproteins containing RdRp at the approximate sizes of 120 kDa, 105 kDa, and 100 kDa, respectively.

Virus-mediated translational shutoff is a strategy used by many RNA viruses to increase the cellular pool of ribosomes for viral protein synthesis and to inhibit antiviral innate responses during infection [1,41]. CrPV infection leads to a significant shutoff of translation at two to three h.p.i. which is the approximate time when IGR IRES translation is stimulated [7]. We previously showed that eIF2α is phosphorylated approximately at this time post infection, however, forced dephosphorylation of eIF2α did not impact host translational shut off or viral protein synthesis [7]. As several studies [14,25,42,43] have shown that eIF2α phosphorylation stimulates IGR IRES translation, we re-examined whether phosphorylation of eIF2α during CrPV infection has an effect on the regulation of 5′UTR and IGR IRES translation. To this end, we inhibited eIF2α phosphorylation by treating S2 cells with double stranded RNAs, dsRNAs, directed against the two known eIF2α kinases in Drosophila, dPERK and dGCN2. Since both dPERK and dGCN2 are required for eIF2α phosphorylation in arsenite-treated S2 cells, dsRNA knockdown efficiencies were assessed indirectly by immunoblotting for eIF2α phosphorylation in arsenite-treated dPERK and dGCN2-depleted S2 cells [44]. Treating cells with both dPERK and dGCN2 dsRNAs but not singly inhibited arsenite-induced eIF2α phosphorylation, which is similar to that observed previously (Figure 5A) [44], thus validating our knockdown conditions. We next determined which kinase is responsible for eIF2α phosphorylation in CrPV-infected S2 cells. We incubated GFP dsRNAs as a control or dPERK or dGCN2 dsRNAs individually or together to S2 cells prior to mock- or CrPV-infection. While CrPV-infected S2 cells treated with control (GFP), GCN2 or PERK dsRNAs resulted in phosphorylation of eIF2α, treating infected cells with both PERK and GCN2 dsRNAs abolished eIF2α phosphorylation (Figure 5A). Thus, our result suggests that both GCN2 and PERK are activated in CrPV-infected S2 cells to induce eIF2α phosphorylation. To determine whether host or viral protein synthesis is perturbed, we monitored protein synthesis by pulse-labelling in CrPV-infected S2 cells treated with control dsRNAs (Figure 5B) or with PERK and GCN2 dsRNAs (Figure 5C). Despite inhibiting eIF2α phosphorylation, host translational shutoff and viral protein synthesis occurred to the same extent and at roughly the same time as in cells treated with the control dsRNAs (Figure 5B,C). Thus, in agreement with previous findings [7], eIF2α phosphorylation is not responsible for host translation shutoff or viral protein synthesis and does not appear to contribute to the temporal regulation of IGR IRES-mediated translation of CrPV ORF2 in CrPV-infected S2 cells.

### 3.6. Premature Translational Shutoff and CrPV IRES Translation

**Figure 6:**
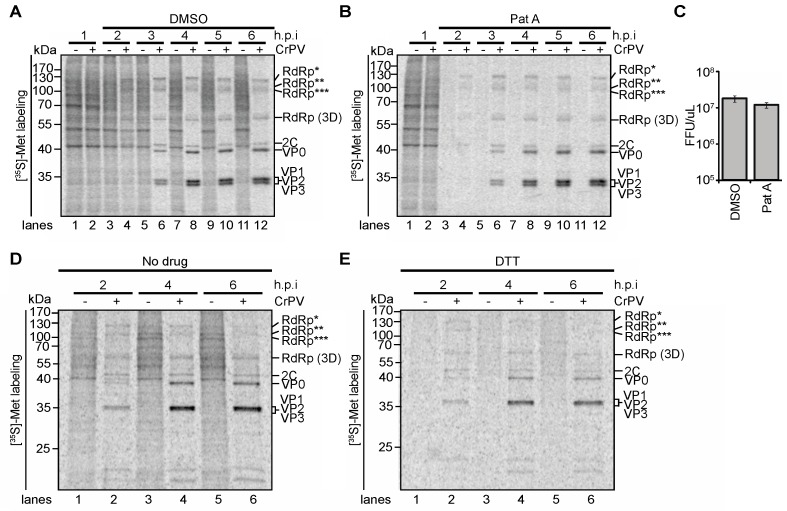
Effects of pateamine A (PatA) and DTT on CrPV protein synthesis. Pulse-labelled protein lysates from mock- or CrPV-infected (MOI 10) S2 cells treated with either (**A**) DMSO; (**B**) 50 nM pateamine A; (**E**) 50 nM DTT; (**D**) or left untreated were resolved on a 12% SDS-PAGE gel. The cells were metabolically labelled with [^35^S]-Met/Cys for twenty minutes at the end of each time point. Shown are representative autoradiographs from at least three independent experiments; (**C**) Viral titers from infected lysates treated with DMSO or PatA after 10 h.p.i. RdRp*, RdRp**, and RdRp*** denote polyproteins containing RdRp at the approximate sizes of 120 kDa, 105 kDa, and 100 kDa respectively.

We next asked whether prematurely repressing host translation can alter the temporal regulation of ORF1 and ORF2 expression in CrPV infected cells. We reasoned that premature inhibition of host translation may stimulate IGR IRES translation earlier in infected cells. Treatment of mammalian cells with the chemical compound, Pateamine A (PatA), which deregulates eIF4A activity, results in a rapid decrease in cap-dependent translation and stimulates IGR IRES-mediated translation [25,45]. We treated mock- or CrPV-infected S2 cells with PatA at one h.p.i. and monitored protein synthesis by pulse-labeling. As expected, PatA inhibited overall host protein synthesis after only one hour of treatment (Figure 6B, compare lane 1 to 3). The effects of PatA continued to abolish translation after 5 h of treatment (Figure 6B, lanes 5, 7, 9 and 11). Compared to DMSO- treated cells (Figure 6A), PatA treatment of CrPV-infected cells resulted in a premature significant shut off of host protein synthesis by 2 h.p.i. (Figure 6B, lane 3). In contrast and interestingly, only the CrPV non-structural proteins could be visibly detected at this time point (Figure 6B, lane 4) and it is not until 3 h.p.i. that synthesis of the structural proteins is detected, which is similar to the time that is observed in DMSO-treated infected cells (Figure 6A,B). Moreover, the extent of viral non-structural and structural protein synthesis and RNA synthesis were similar between DMSO- and PatA-treatments over the course of infection (Figure 6C). Finally, PatA treatment did not significantly affect intracellular CrPV yield, thus suggesting that premature translational shut off in S2 cells does not affect viral replication (Figure 6C). To ensure these results are not specific to PatA, we prematurely inhibited cellular translation with DTT, a treatment that induces eIF2α phosphorylation and also known to stimulate IGR IRES translation (Figure 6D,E). Like PatA treatment, we observed no differences in the timing or extent of viral non-structural and structural protein synthesis in untreated and DTT-treated infected cells (Figure 6D,E). Thus, forced premature inhibition of protein synthesis does not disrupt the temporal regulation of IGR IRES-mediated translation.

### 3.7. Non-Structural Viral Proteins Promote Expression of Structural Viral Proteins

**Figure 7:**
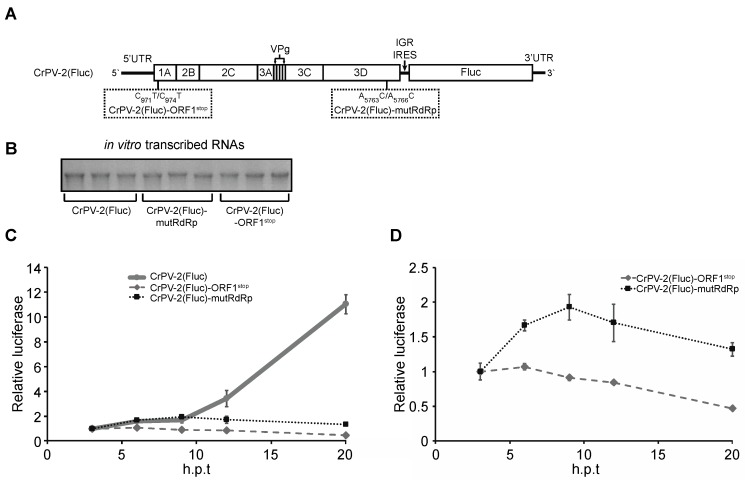
Non-structural viral proteins promote IGR IRES translation. (**A**) Schematics of the CrPV2(Fluc), CrPV2(Fluc)-ORF1^stop^ and CrPV2(Fluc)-mutRdRp replicons; (**B**) *In vitro* transcribed CrPV2(Fluc), CrPV2(Fluc)-ORF1^stop^ and CrPV2(Fluc)-mutRdRp RNAs that were used in the transfections as shown in (**C**,**D**); (**C**) Firefly luciferase activities from S2 cells transfected with CrPV2(Fluc), CrPV2(Fluc)-ORF1^stop^ and CrPV2(Fluc)-mutRdRp RNAs at different time points transfection. The results are normalized to its respective three-hour post transfection (h.p.t); (**D**) Results are from (**C**) but only showing CrPV-2(Fluc)-ORF1^stop^ and CrPV(FLuc)-mutRdRP. Shown are results from an average of three independent experiments (±s.d.).

Because IGR IRES-mediated translation is not perturbed by premature inhibition of cellular translation or transcription, we next asked if one or more non-structural viral proteins may be responsible for the regulation of IGR IRES activity. To test this, we created a CrPV replicon (CrPV2(Fluc)) by replacing the ORF2 structural proteins of the CrPV-2 infectious clone with firefly luciferase [27]. Thus, firefly luciferase expression in this replicon is under the control of the IGR IRES (Figure 7A). To address whether expression of the non-structural proteins affects IGR IRES translation, we generated another replicon (CrPV2(Fluc)-ORF1^stop^), which contains two stop codons within the most N-terminal protein of ORF1, CrPV1A, and thus prevents ORF1 expression (Figure 7A). The *in vitro* transcribed replicons (Figure 7B) were transfected into S2 cells and luciferase activity was measured at different time points after transfection (hours post transfection (h.p.t)). Whereas (CrPV2(Fluc)-ORF1^stop^) RNA transfections led to relatively low luciferase activity, CrPV2(Fluc) RNA transfections resulted in a significant increase in luciferase activity, especially between 12 and 20 h after transfection (Figure 7C). These results suggest that expression of the non-structural proteins contributes to the increase in IGR IRES translation.

The increase in luciferase activity observed from CrPV2(Fluc) transfections is likely due to contributions to an increase in IGR IRES translation and replication. To uncouple these effects, we mutated two conserved residues (D1620A and D1621A) within the RdRp that have been shown to be important for replicase activity (CrPV2(Fluc)-mutRdRp) [46]. Transfection with (CrPV2(Fluc)-mutRdRp) RNA did not lead to an increase in luciferase activity as compared to the CrPV2(Fluc) transfection (Figure 7B), suggesting that replication of the CrPV replicon contributes significantly to the increase in luciferase activity from CrPV2(Fluc) transfections. However, a careful comparison of luciferase activities between (CrPV2(Fluc)-mutRdRp) and (CrPV2(Fluc)-ORF1^stop^) transfections (Figure 7D) showed that there is a reproducible two-fold increase in luciferase activity of (CrPV2(Fluc)-mutRdRp) over (CrPV2(Fluc)-ORF1^stop^) transfections, indicating that IGR IRES activity is stimulated similar to that observed with the reporter construct assays and ribosome profiling (Figure 2, Figure 3A,B and Figure 7D). These results further suggest that expression of a non-structural protein(s) contribute to the increase in IGR IRES activity.

## 4. Discussion

**Figure 8:**
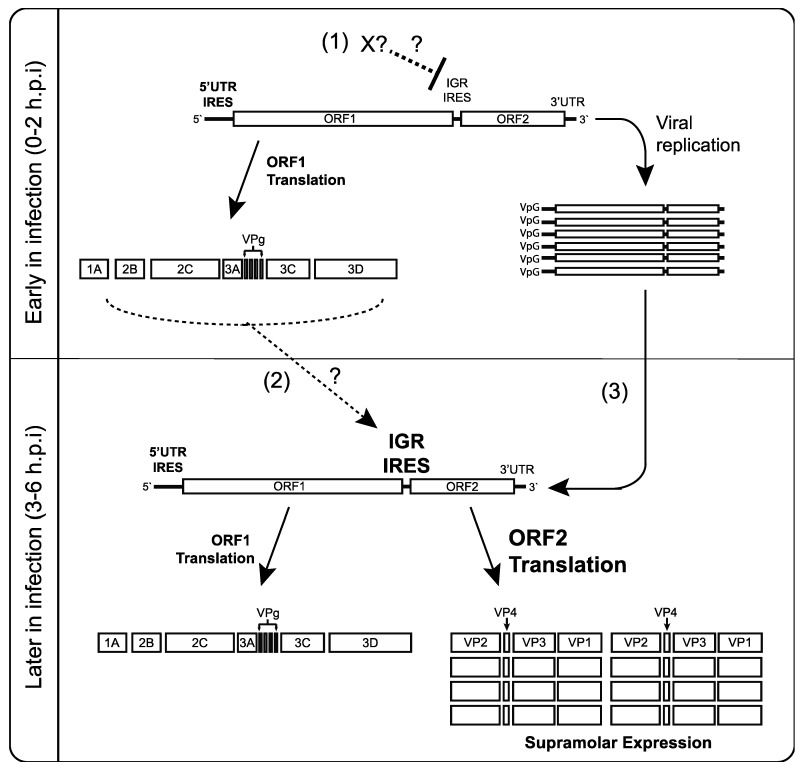
Model of CrPV 5′UTR IRES and IGR IRES-dependent translation during infection. Early in infection (0–2 hpi), 5′UTR IRES-dependent translation predominates to produce viral non-structural proteins such as the 3D RNA-dependent RNA polymerase, the 3C-like protease and helicase. (1) IGR IRES translation may be inhibited directly or indirectly by a yet unknown factor; (2) At 3–4 hpi, non-structural viral proteins may directly or indirectly stimulate IGR IRES translation (dashed arrow), leading to viral structural protein expression; (3) Replication of the viral genome enhances structural protein expression by providing more translatable CrPV genomes and/or stimulating IGR IRES activity. In effect, the temporal regulation of viral protein synthesis via IRES control allows viral replication to occur prior to structural protein synthesis.

Dicistroviruses utilize a strategy whereby distinct IRESs regulate translation of non-structural and structural protein ORFs. While it has been well established that the viral structural proteins are produced in supramolar excess over non-structural viral proteins [7,8,9], the significance and the mechanisms underlying this phenomenon have not been investigated in detail. Here, we demonstrate that translation of the two ORFs via the 5′UTR and IGR IRESs are temporally regulated (Figure 8). Using a series of biochemical and molecular assays, we show conclusively that there is a reproducible delay, albeit slight, in IGR IRES translation compared to 5′UTR IRES translation during CrPV infection (Figure 1, Figure 2 and Figure 3). We also showed that this temporal regulation is not dependent on the repression of host transcriptional or translational inhibition, cellular processes that are perturbed during CrPV infection (Figure 4, Figure 5 and Figure 6). Using CrPV(Fluc) replicons to decouple viral translation from viral replication, we showed that the increase in IGR IRES translation is partly dependent on viral non-structural proteins (Figure 7). We propose that CrPV utilizes a dual IRES translational control strategy to ensure that optimal expression of viral non-structural proteins occurs prior to synthesis of viral structural proteins (Figure 8), and as a result, this leads to the temporal regulation of viral replication and virus assembly during infection.

Regulation of IRES translation is key for some virus infections. For instance, poliovirus uses a strategy via temporal cleavage of host factors such as the translation factors eIF4G and poly (A) binding protein and IRES-trans-acting factors, Poly(rC) binding protein 2 and polypyrimidine tract binding protein 1, to regulate the switch from host translation to IRES-mediated protein synthesis as well as the switch from viral translation to replication [3,47,48,49]. In this study, we examined whether the distinct temporal regulation of non-structural and structural protein synthesis during CrPV infection may be driven by the shutoff of host translation and the different factor requirements between IGR IRES and 5′UTR dependent translation [13,14,24]. Surprisingly, attempts to disrupt overall host translation by either inhibiting eIF2α phosphorylation or affecting eIF4A activity during infection did not affect the timing of 5′UTR and IGR IRES translation (Figure 5 and Figure 6). Although it has been shown that CrPV IGR IRES translation is stimulated when eIF2α is phosphorylated or eIF4A activity is disrupted [14,25,42], it is clear that indirect effects through translational shutoff are not a prerequisite for IGR IRES stimulation during CrPV infection.

One possible explanation is that there may be a more direct effect on IGR IRES translation during infection: (i) IGR IRES translation may be inhibited at early time points during infection or (ii) IGR IRES translation may be activated at later times. Our data suggest that besides RdRp, one or more non-structural proteins contribute to the increased structural protein synthesis during CrPV infection (Figure 7). Specifically, a reproducible two-fold increase in luciferase activity was noted when comparing a mutant replicon that is defective in replicating (CrPV2(Fluc)-mutRdRp) to a mutant replicon that expresses no non-structural viral proteins (CrPV2(Fluc)-ORF1^stop^) (Figure 7). These results suggest that non-structural proteins may act directly or indirectly to regulate IGR IRES translation during CrPV infection, potentially via structural conformations of the IGR IRES and/or affecting ribosome recruitment (Figure 7). A possible target is the ribosome. It has been shown that depletion of a subset of ribosomal proteins results in inhibition of IRES-dependent but not cap-dependent translation [29,50,51,52], suggesting that heterogeneity of ribosomes may have an effect on IRES translation. For example, depletion of a subset of ribosomal proteins inhibits IGR IRES translation and infectivity by a related dicistrovirus Drosophila C virus [29,50,51]. Interestingly, cells depleted of RACK1, a component of the 40S subunit, leads to inhibition of 5′UTR IRES but not IGR IRES translation during infection, thus further supporting that the two CrPV IRESs are differentially regulated at the ribosome level [53]. Thus, it remains to be examined whether specific post-translational modifications or depletion of key ribosomal proteins during CrPV infection may contribute to the temporal regulation of IRES translation.

As described earlier, a number of viruses, including poliovirus, recruit ITAFs to facilitate viral translation [54,55]. It is possible that a similar phenomena is occurring under CrPV infection. However, there have yet to be any reports of host (or viral) factors other than the ribosome that bind to the IGR IRES. Moreover, given the compact structure of the IRES, it is difficult to imagine a trans-acting factor binding to the IGR IRES [16,20,21,22,23]. Nevertheless, if an ITAF is involved, our data suggest it is not likely a newly transcribed cellular RNA or translated host protein that mediates IGR IRES-dependent translation during infection (Figure 4 and Figure 6).

A key finding from our studies is that replication of the CrPV genome is necessary for the dramatic increase in firefly luciferase, which monitors IGR IRES translation in the replicon system (Figure 7). In contrast, despite the significant increase in viral RNA during CrPV infection, 5′UTR IRES-mediated translation is not stimulated (Figure 1), thus underscoring the differential regulation of the distinct IRESs during CrPV infection. Because IGR IRES-mediated translation is stimulated during infection by approximately 3–4 fold (Figure 3) [25], the supramolar expression of structural proteins is likely due to a sum of both the effects of the increase in replicating CrPV viral RNA genome and the stimulation of IGR IRES translation. Alternatively, IGR IRES translation may be stimulated even more within viral progeny genomes. It has been reported that newly transcribed vaccinia RNAs during infection are preferentially translated during infection [56]. Thus, a similar scenario may be occurring in CrPV replicating genomes that results in preferential IGR IRES translation, a hypothesis that warrants further examination.

In a recent report, Wu *et al.* (2014) demonstrated that Pelo is required for enhanced structural protein synthesis during dicistrovirus infection but does not affect IGR IRES translation [57]. Pelo is the *Drosophila* homolog for Dom34, which is responsible for the recycling of stalled 80S ribosomes on mRNAs [58]. Wu *et al.* (2014) speculated that the action of Pelo during infection provides dicistrovirus genomes greater access to ribosomes for high level synthesis of viral structural proteins [58]. Nevertheless, in cells depleted of Pelo, dicistrovirus structural proteins are still expressed in supramolar excess over non-structural proteins [57], thus ribosome recycling is likely not the sole reason for structural protein synthesis that is observed late in infection.

## 5. Conclusions

Many viruses use a strategy to coordinate temporal expression of non-structural and structural proteins [26]. We propose CrPV uses an IRES-based strategy to initiate expression of non-structural proteins for viral translation and replication and delay of expression of the structural proteins during infection, which may be a strategy generalized within the *Dicistroviridae* family.

## Supplementary Materials

The following is available online at www.mdpi.com/1999-4915/8/1/25/s1, Figure S1: CrPV protein synthesis by immunoprecipitation analysis.
