# Supplementary material for: Temporal Regulation of Distinct Internal Ribosome Entry Sites of the Dicistroviridae Cricket Paralysis Virus
Source: Viruses. 2016 Jan 19;8(1):25. doi: 10.3390/v8010025 (PMC4728584; doi:10.3390/v8010025)
Supplement: Supplementary file 1 [file viruses-08-00025-s001.pdf]

Supplementary Materials: Temporal Regulation of Distinct Internal Ribosome Entry Sites of the *Dicistroviridae* Cricket Paralysis Virus

Anthony Khong, Jennifer M. Bonderoff, Ruth V. Spriggs, Erik Tammpere, Craig H. Kerr, Thomas J. Jackson, Anne E. Willis and Eric Jan

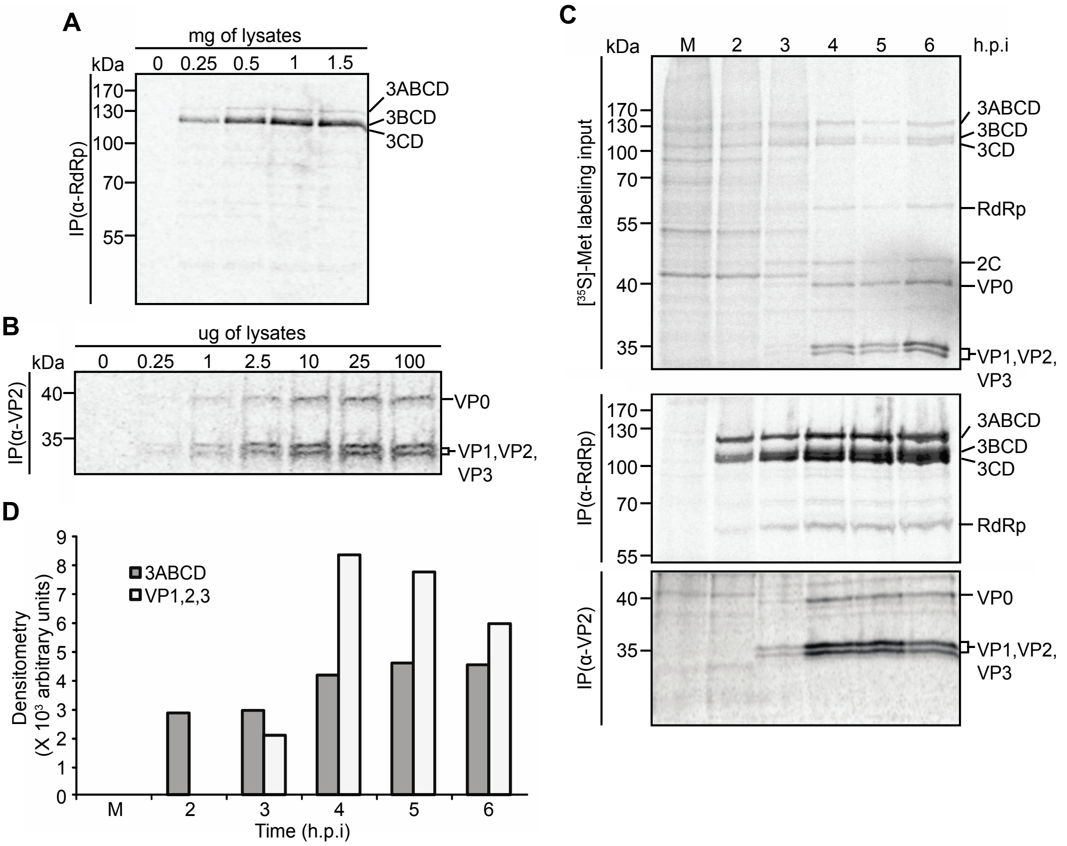

Figure S1. CrPV protein synthesis by immunoprecipitation analysis. Increasing amounts of pulse labelled protein lysates from CrPV-infected S2 cells (MOI 10, six h.p.i) were immunoprecipitated with a fixed amount of (**A**) α-RdRp or (**B**) α-VP2 antibodies. Pulldowns were resolved on an 12% SDS-PAGE; (**C**) 0.25 mg and 2.5 μg of protein lysates isolated from mock- or CrPV-infected (MOI 10) cells for the indicated times (hpi) were immunoprecipitated with α-RdRp or α-VP2 antibodies respectively; (**D**) Raw densitometric quantitation of immunoprecipitated pulse-labelled RdRp* protein and structural proteins VP1, VP2, and VP3 from (**C**). Cells were metabolically labelled with [^{35}S]-Met/Cys for one hour prior to the end of each time point. Shown are representative autoradiographs from 2 experiments. RdRp*, RdRp**, and RdRp*** denote polyproteins containing RdRp at the approximate sizes of 120 kDa, 105 kDa, and 100 kDa respectively.

© 2016 by the authors; licensee MDPI, Basel, Switzerland. This article is an open access article distributed under the terms and conditions of the Creative Commons by Attribution (CC-BY) license (http://creativecommons.org/licenses/by/4.0/).
